# Microstructure of CEM II/B-S Pastes Modified with Set Accelerating Admixtures

**DOI:** 10.3390/ma14216300

**Published:** 2021-10-22

**Authors:** Jan Pizoń, Beata Łaźniewska-Piekarczyk

**Affiliations:** Faculty of Civil Engineering, Silesian University of Technology, 44-100 Gliwice, Poland; beata.lazniewska-piekarczyk@polsl.pl

**Keywords:** cement, accelerating admixtures, concrete, microstructure, SEM

## Abstract

The presented paper aims to describe the influence of accelerating admixtures on the properties and microstructure of cement pastes and mortars. Blended slag cement CEM II/B-S containing two different clinkers (differing amounts of siliceous and aluminous phases) and four types of accelerators (calcium nitrate, sodium hydroxide, cement kiln dust, and crystal seeds) were used in research. Compressive strength tests (after 12, 24, 48 h of curing), Scanning Electron Microscope (SEM) observations together with an Energy Dispersive Spectroscopy (EDS) analysis, Mercury Intrusion Porosimetry (MIP) tests, and X-ray diffraction (XRD) analysis were conducted. Results have shown that SEM and EDS examination of the microstructure of cement pastes modified with accelerating admixtures at the observed points did not reveal differences that would be sufficient to explain the changes in compressive strength. Still, the increase in amorphous phase content indicates a faster hydration reaction rate for all pastes modified with accelerating admixture. It is backed up also by lower non-hydrated compounds content. All admixtures accelerate the hydration reaction of calcium silicate phases of cement, but only NaOH and cement kiln dust (CKD) influence the aluminate phase reaction rate. The pore volume is independent of the clinker type, while the pore size distribution is not.

## 1. Introduction

The presented paper describes the influence of accelerating admixtures on the properties and microstructure of cement pastes and mortars. The publications [1,2] describe the microstructure of cement pastes and mortars in the range of high C_3_A cement [3]. The microstructure was analyzed in the form of microscopic observations without XRD or porosimetry analysis. This paper is a continuation and extension of previous research. This paper also gives a comparison of high and normal C_3_A types of cement. Accelerators involved in research were: modern accelerator providing heterogeneous nucleation seeds; cement kiln dust (CKD), used because of its great fineness and similarity in composition to Portland cement (and possibility to act as nucleating seeds as well); sodium hydroxide, because of its proven activating effect on alkali activated slag (AAS); and calcium nitrate as a reference, well-known, and commonly used accelerator. The most relevant aspect of the conducted research is the observation of the effects of CKD and modern accelerator on microstructure of cement pastes.

Concrete is one of the primary building materials. Its main ingredient is cement, for which, production in 2016 exceeded 4.2 billion tons globally [4]. The amount of cement produced has been steady in recent years, reaching about 4.1 billion tons in 2019 as well [4]. Cement production has a significant impact on the environment. It is estimated that this sector is responsible for about 5–8% of anthropogenic CO_2_ emissions. [5]. Emissions are caused mainly by fuel combustion and CaCO_3_ decomposition [6]. Therefore, it is crucial to advance in cement and concrete technology in any way that allows decreasing this emission [7]. One of the possibilities is to speed up construction works by accelerating setting and hardening of cement [8,9].

Mineral additives and chemical admixtures are commonly used in cement and concrete technology. One of the most widely used mineral additives is ground granulated blast-furnace slag (GGBFS). The result of its reaction with water is primarily the C-S-H (calcium silicate hydrate) phase and other phases that may also be found in Portland cement paste [10].

During the initial period of hydration of cements containing slag, Portland clinker phases are primarily responsible for strength development. Later slag activity increases, increasing in the C-S-H phase content [11,12]. The hydration process of blast furnace slag in cement paste begins by reacting with sodium and potassium hydroxides and, almost simultaneously, with calcium hydroxide [10,13,14,15]. The microstructure of hardened pastes of cement containing slag is denser than OPC (ordinary Portland cement) [16,17]. These contain much more C-S-H phase and much less calcium hydroxide [10,18]. The finer pore structure is produced while the GGBFS is incorporated in the cement [19]. Hydrated calcium aluminates are also formed since many aluminum ions are present in the slag [16]. The composition of the C-S-H phase formed by the hydration of cement containing slag has a higher Si/Ca molar ratio than OPC [18,20]. The strength can significantly affect cement with high C_3_A (tricalcium aluminate) content during the initial hardening period [21].

Set and hardening accelerating admixtures are often used to shorten the period to release forms in prefabrication plants and enhance early compressive strength. Those effects are widely known [8,10,22,23]. However, there are few studies on the microstructure of cement composites containing these admixtures. This paper tries to fill this gap. Especially, there are few descriptions of the microstructure of composites containing modern accelerating admixtures with nucleation seeds for concrete.

The effect of the setting and hardening accelerating admixtures on the microstructure development depends on their chemical composition. Calcium nitrite and calcium nitrate are commonly used as accelerating admixtures for concrete. Both contain calcium cations like phases of Portland clinker. The effect of its usage—shortening initial setting time and increasing early-stage compressive strength—is widely known [22,24,25]. These admixtures can act as a nucleation seed and cause an accelerated reaction of calcium silicates (mainly C_3_S—tricalcium silicate) with water [9]. The addition of calcium nitrate results in a cement paste microstructure with similar total porosity and more gel pores [24]. At the early stage, the calcium nitrate promotes the hydration of C_3_S and the formation of C-S-H phase’s silicate chains, thus it increases the specific surface area [26]. It is also stated that calcium nitrate and nitrite are embedded into the AFm (alumina, ferric oxide, tri-sulfate) and AFt (alumina, ferric oxide, mono-sulfate) and is affecting the ratio of AFm/Aft. In consequence, it affects the specific volume of hardened paste [27].

Cement kiln dust (CKD) is a by-product of Portland clinker production. It is a very fine, powdered, grey material with micron-sized grains. Its composition highly depends on the raw materials and fuel used in the cement’s manufacture [28]. CKD contains significant amounts of sodium and potassium, which accelerates the strength development of slag cement by increasing the rate of slag hydration [29]. CKD has a large specific surface area of about 4600–14,000 cm^2^/g [28], so that the dissolution rate of its components in water is notable. CKD also contains chlorides and calcium carbonate. These components are used as admixtures to accelerate cement setting and hardening [9]. The reactivity of CKD is primarily dependent on its CaO content [30,31]. According to some sources, a small addition of CKD (5–15%) can increase the strength, shorten the initial and final setting times and improve the porosity pattern by increasing the number of gel pores [32,33]. The excellent strength development was observed in research [34,35], in which the microstructure was also examined. It was concluded that the microstructure of CKD containing cement mortars is more uniform, denser, and less porous.

Sodium hydroxide dissolved in the batch water causes an increase in pH, resulting in faster hydration of the slag [9,36,37]. The effect of increased strength with NaOH occurs only during the initial hardening phase [3,9]. This compound is also commonly used as an alkali activator for alkali-activated slag mortars (AAS), containing GGBFS [37,38]. Because of those properties, it was incorporated into the present research. In terms of microstructure there, is little information in the literature. The addition of sodium hydroxide forms a C-S-H phase with a smaller surface area than that formed without the hydroxide [9]. There was no effect of sodium hydroxide on the total porosity of pastes [9,39].

Modern setting and hardening accelerating admixtures mainly have a physical effect by introducing additional micro- or nanoparticles into the batch water, which play the role of heterogeneous nucleation seeds (C-S-H seeds, crystallization seeds) [40]. Such particles are synthetic C-S-H phase—the main product of hydration of the cement that is dispersed in cement paste [41,42]. As a result, hydration products are formed not only on the surface of cement grains, but also in the spaces between them [42,43,44]. This effect is visible also in the term of refinement of porosity pattern [43,45]. The microstructure of the cement matrix was reported to be much denser and finer after six [46] and eight hours of curing [47]. Hardened pastes with these admixtures obtain a denser microstructure faster [48,49]. It was stated that the microstructure of hydration products was changed using C-S-H seed admixture [50]. The same work reports potential problems with the water demand of cement modified with nanoparticles and with decreased compressive strength. It was also reported in another manuscript at w/c = 0.3. In the case of higher w/c ratios, the effect disappeared [45].

## 2. Materials and Methods

### 2.1. Types of Cements

**Figure 1:**
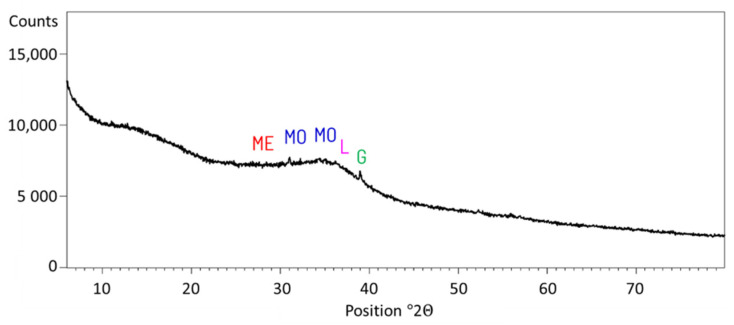
XRD analysis of GGBFS. ME—Merwinite (Ca_3_Mg(SiO_4_)_2_); MO—Monticellite (CaMgSiO_4_); L—Larnite (β-C_2_S,Ca_2_SiO_4_); G—Gelhenite (Ca_2_Al(AlSiO_7_)).

The tests were conducted with blended slag cement (CEM II/B-S), containing 35% of ground granulated blast furnace slag (GGBFS). The cement were blends of Portland clinker, GGBFS, and anhydrite as a set regulator. Two types of Portland clinkers (C1, C2) differing in phase composition were used. The choice of such clinkers is supported by the differences in their behavior mainly in the initial hydration stage. More C_3_A phase containing cement sets and hardens faster [8,21]. Besides that, different admixtures react with different cement phases and their efficiency depends on it [9]. Characteristics of both clinkers are given in Table 1. The anhydrite amount was set for obtaining 2% of SO_3_ in the resulting cement. The chemical composition of anhydrite is given in Table 1. GGBFS used in the research was obtained from one of the polish ironworks. The main characteristics of GGBFS required by EN 15167-1—the reactivity indices are greater than required by EN—were after 7 days 62.8% (required by the standard is 45%) and after 28 days 88.3% (required by the standard is 70%). The amorphous phase content of 98.5% is also higher than 67% required by EN 197-1. The chemical composition of slag is given in Table 1. The X-ray diffraction (XRD) analysis was conducted for GGBFS. The result is given in Figure 1.

### 2.2. Accelerating Admixtures

**Figure 2:**
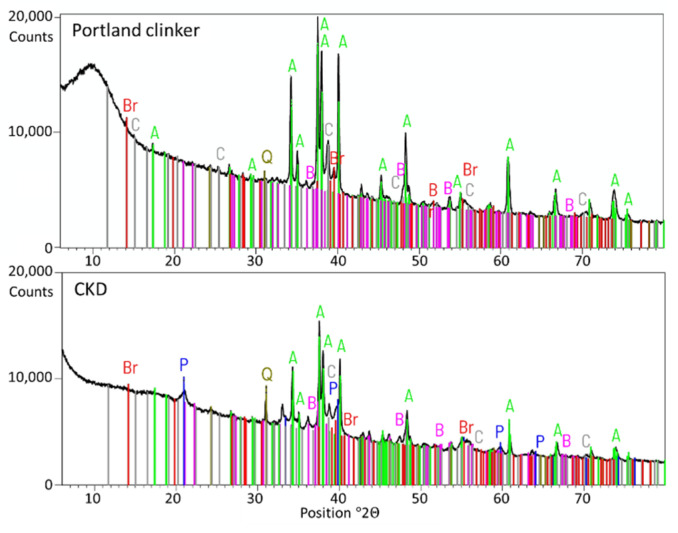
XRD analysis of CKD and the Portland clinker from which production it was obtained. A—Alite, tricalcium silicate (C_3_S, Ca_3_SiO_5_); B—Belite, dicalcium silicate (α-C_2_S,Ca_2_SiO_4_); C—tricalcium aluminate (C_3_A, Ca_3_Al_2_O_6_); Br—Brownmillerite, tetracalcium aluminoferrite (C_4_AF, Ca_2_(Al,Fe)_2_O_5_); Q—quartz (SiO_2_); P—portlandite (Ca(OH)_2_).

Four accelerating agents were involved in the research:

- 20% sodium hydroxide (NaOH) solution. The solution was introduced to the batch water to obtain 5% NaOH according to cement’s mass.
- 20% calcium nitrate tetrahydrate (Ca(NO_3_)_2_·4H_2_O). The solution was added to reach 2% of calcium nitrate for the cement’s mass (symbol CN).
- Cement kiln dust (CKD)—obtained from one of the polish cement plants. CKD was added as an ingredient of the cement. The amount was established as 5% of total cement’s mass. The chemical composition of CKD is given in Table 2. The X-ray diffraction (XRD) analysis was conducted for CKD and Portland clinker from which manufacture the CKD was derived. The results are given in Figure 2. The similarity of constituents described in the Introduction section is visible.
- Available on market modern setting and hardening accelerating admixture containing crystal seeds in form of C-S-H nanoparticles (symbol CS).

Cement mortars for compressive strength tests were prepared with ingredients listed above in the proportions stated in Table 3. The composition of cement pastes for Scanning Electron Microscope (SEM), Energy Dispersive Spectroscopy (EDS), X-ray diffraction (XRD), porosimetry, and initial setting time tests is given in Table 4.

### 2.3. Methods

Compressive strength tests, SEM observations, EDS analysis, Mercury Intrusion Porosimetry (MIP) tests, and XRD analysis were conducted.

Mortar samples with dimensions 160 mm × 40 mm × 40 mm for compressive strength tests were prepared according to EN 196-1. The samples were broken into halves, which were tested. Loading area was 40 mm × 40 mm according to EN 196-1. The samples were cured for 12 h in a climatic chamber at a temperature of 20 ± 1 °C and relative humidity of 60%. After this time, the samples were demolded and then the samples were cured in water at 20 ± 1 °C. Compressive strength testing was performed after 12 h and after 1 and 2 days of curing.

Samples of cement pastes intended for microstructure tests were prepared according to PN-EN 196-3 standard with a water-cement ratio of 0.5. The samples were prepared in the form of tiny prisms with dimensions of 5 mm × 5 mm × 25 mm (SEM + EDS) and 10 mm × 10 mm × 30 mm (MIP, XRD). The samples were properly compacted to avoid unintended pores occurrence. The samples were cured for 48 h, covered with a wet cloth, at a temperature of 20 ± 1 °C. One hour before testing, the samples were dried at a temperature of 40 °C to avoid the microstructure change connected to high temperature—possible dehydration of gypsum—and hence the change of microstructure. A similar procedure was described by [51,52].

Microscopic observations of the fractures of samples were performed. The investigations were carried out using a SEM equipped with an EDS analyzer allowing the determination of the chemical composition of selected phases in the micro-areas.

The center sections were cut from 10 mm × 10 mm × 30 mm cuboid samples and placed in a porosimeter to determine the volume of air pores. The method used was Mercury Intrusion Porosimetry (MIP).

The samples intended for XRD testing were those formed by grinding the samples tested in the SEM. The tests were performed using the PANalytical Empyrean instrument using filtered cobalt radiation, configured with Pixcel detector. Phase composition identification was performed according to the International Centre for Diffraction Data PDF-4+ database, version 2016. Quantitative results were obtained using the Rietveld refinement as used for testing multicomponent materials. The sample for quantitative phase analysis was prepared as a mixture of ground cement paste sample and powdered corundum (certified Standard Reference Material No. 676a, manufactured at the National Institute for Standards and Technology, Gaithersburg, MD, USA). Due to the significant content of the amorphous phase in the cement paste samples (originating from the hydration product—C-S-H phase and GGBFS), the content of non-crystalline constituents was determined by subtracting the content; of identified and determined contributions of crystalline phases from the total.

SEM + EDS and MIP studies were performed for cements containing C1 and C2 clinkers. XRD examination was performed only for cement containing C1 clinker.

## 3. Results and Discussion

### 3.1. Compressive Strength

**Figure 3:**
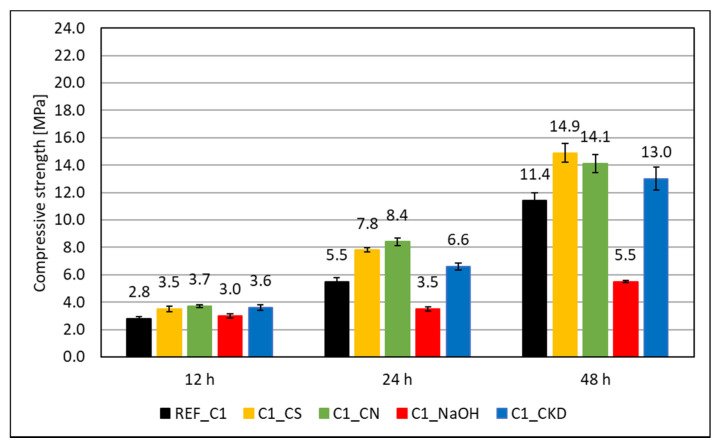
Compressive strength of mortars made with clinker C1 modified with accelerating admixtures.

**Figure 4:**
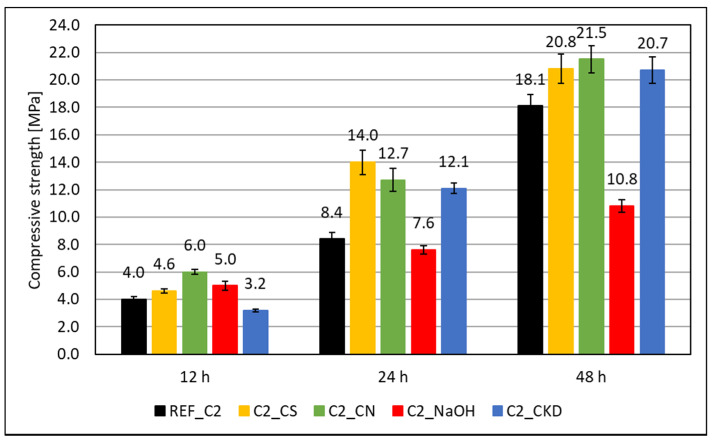
Compressive strength of mortars made with clinker C2 modified with accelerating admixtures.

The results of the compressive strength test are given in Figure 3 and Figure 4. In the initial period of hardening—up to 48 h—the compressive strength of mortars made of Portland cement slag CEM II/B-S containing C2 clinker is higher by 30%, 35%, and 38% after 12, 24, and 48 h, respectively, than that of one containing C1 clinker. This was due to the higher content of tricalcium silicate in the C2 clinker. It is claimed that, in the initial period, the cement containing a lot of C_3_S and about 10% C_3_A provides the highest strength, especially in the case of high acid slags [10].

The effectiveness of the hardening accelerating admixtures also depends on the phase composition of the clinker used to manufacture the cement. In the initial period (up to 48 h), for Portland slag cement CEM II/B-S made from clinker C1, containing more C_3_A, the effectiveness of admixtures containing crystallization seeds (CS) and calcium nitrate (CN) is similar. For cement made from C2 clinker, with higher total silicate content (dicalcium silicate–C_2_S and tricalcium silicate–C_3_S), calcium nitrate has greater efficiency. Similar results were obtained by other authors [25,53,54,55]. CKD also increases the compressive strength of mortars at an early age. Its effectiveness does not depend on the phase composition of the clinkers. It is also lower than crystallization seeds and calcium nitrate. Sodium hydroxide (NaOH) significantly increases the compressive strength of mortars after 12 h of curing, but decreases it after a longer curing time, which has been known for a long time [9].

### 3.2. Scanning Electron Microscopy and Energy Dispersive Spectroscopy

**Figure A1:**
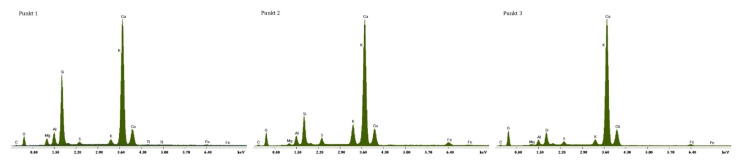
EDS analysis results for non-modified (REF_C1) cement paste made with CEM II/B-S manufactured with clinker C1. 1—C-S-H phase, 2—ettringite, 3—portlandite.

**Figure A2:**
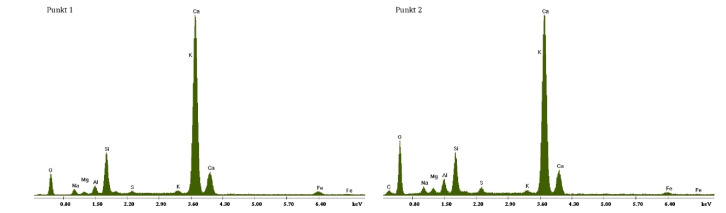
EDS analysis results for non-modified (REF_C2) cement paste made with CEM II/B-S manufactured with clinker C2. 1—C-S-H phase, 2—portlandite.

**Figure A3:**
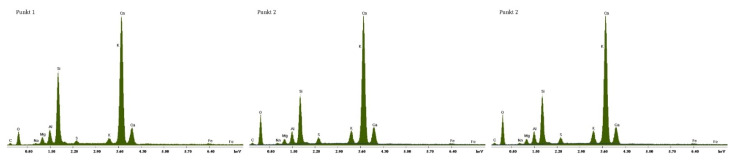
EDS analysis results for cement paste made with CEM II/B-S manufactured with clinker C1, modified with crystallization seeds (CS). 1—C-S-H phase, 2—ettringite, 3—calcite.

**Figure A4:**
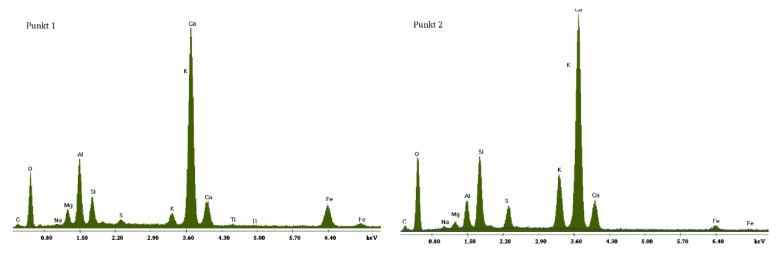
EDS analysis results for cement paste made with CEM II/B-S manufactured with clinker C2, modified with crystallization seeds (CS). 1—AFm phase, 2—ettringite.

**Figure A5:**
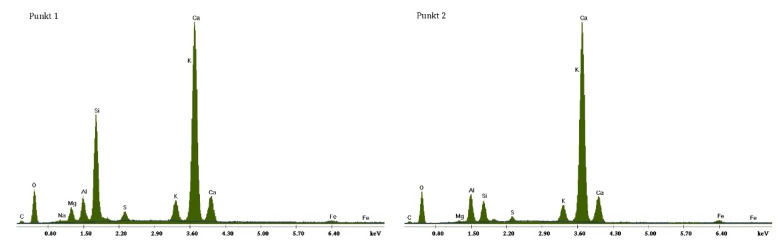
EDS analysis results for cement paste made with CEM II/B-S manufactured with clinker C1, modified with calcium nitrate (CN). 1—C-S-H phase, 2—portlandite.

**Figure A6:**
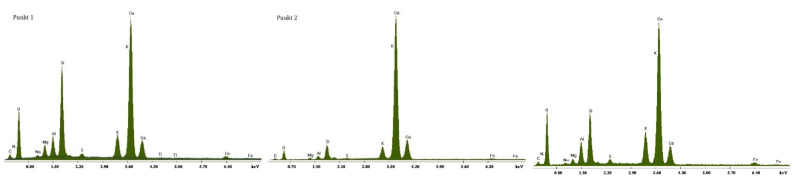
EDS analysis results for cement paste made with CEM II/B-S manufactured with clinker C2, modified with calcium nitrate (CN). 1—C-S-H, 2—CaO, 3—portlandite.

**Figure A7:**
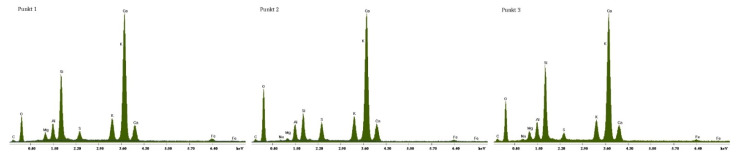
EDS analysis results for cement paste made with CEM II/B-S manufactured with clinker C1, modified with sodium hydroxide (NaOH). 1—C-S-H phase, 2—ettringite, 3—portlandite.

**Figure A8:**
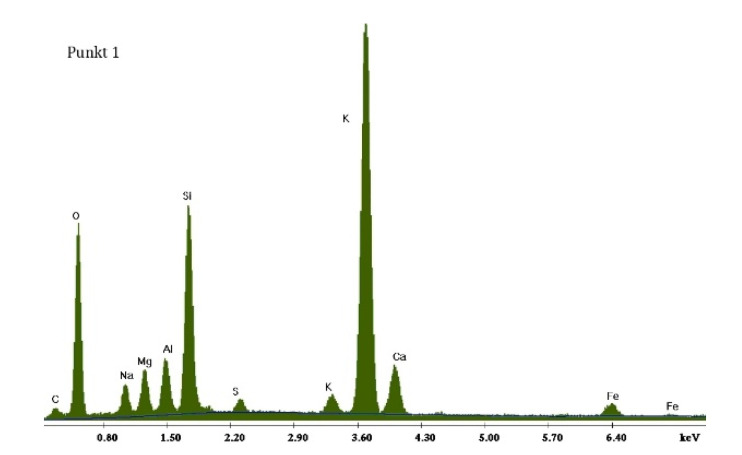
EDS analysis results for cement paste made with CEM II/B-S manufactured with clinker C2, modified with sodium hydroxide (NaOH). 1—C-S-H phase.

**Figure A9:**
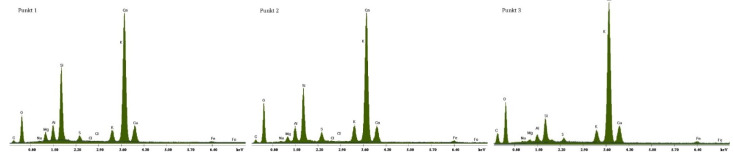
EDS analysis results for cement paste made with CEM II/B-S manufactured with clinker C1, modified with cement kiln dust (CKD). 1—C-S-H phase, 2—ettringite, 3—calcite.

**Figure A10:**
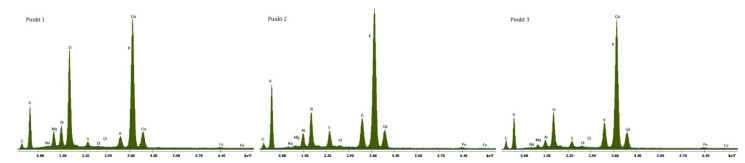
EDS analysis results for cement paste made with CEM II/B-S manufactured with clinker C2, modified with cement kiln dust (CKD). 1—C-S-H phase, 2—ettringite, 3—calcite.

After 48 h of curing, the compressive strength of the mortars varies with the type of clinker and admixture used to accelerate setting and hardening. Therefore, an attempt was made to explain those differences by observing the microstructure of the grouts made from them under a scanning electron microscope (SEM). Respective EDS spectral point analysis graphs are presented in Appendix A (Figure A1, Figure A2, Figure A3, Figure A4, Figure A5, Figure A6, Figure A7, Figure A8, Figure A9 and Figure A10).

#### 3.2.1. Non-Modified Cement Pastes

**Figure 5:**
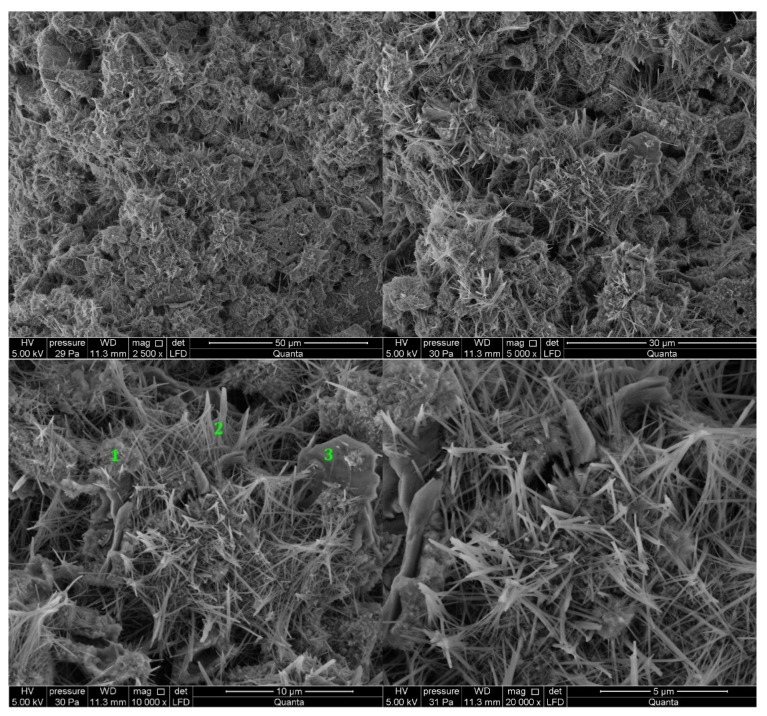
SEM images for non-modified (REF_C1) cement paste made with CEM II/B-S manufactured with clinker C1. 1—C-S-H phase, 2—ettringite, 3—portlandite.

**Figure 6:**
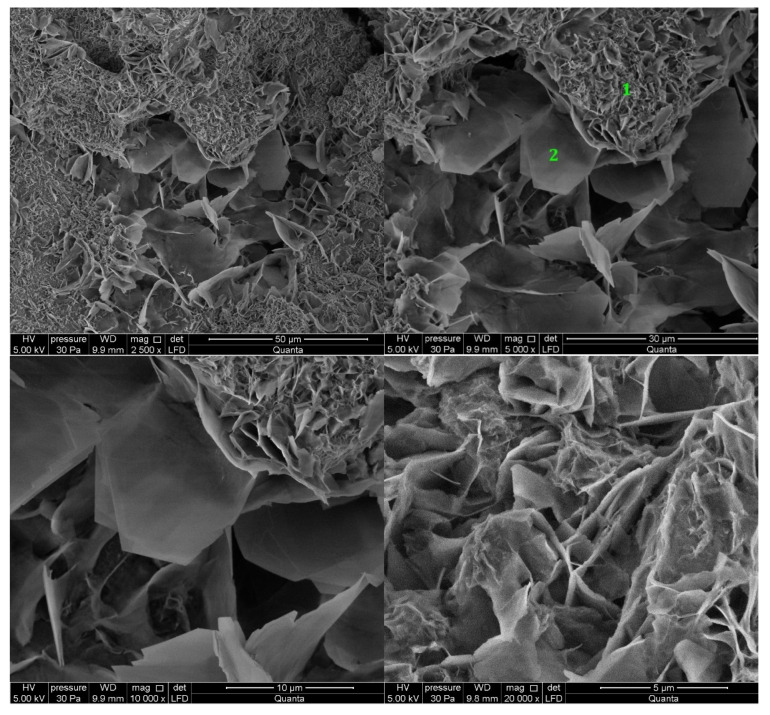
SEM images for non-modified (REF_C2) cement paste made with CEM II/B-S manufactured with clinker C2. 1—C-S-H phase, 2—portlandite.

Microscopic images of cement paste samples of Portland slag cements CEM II/B-S containing clinkers C1 and C2 without the addition of modifiers are shown in Figure 5 and Figure 6, respectively. C2 clinker cement paste contains more C-S-H phase and portlandite at the tested points. The C-S-H phase is found in the form of a honeycomb [10,16]. On the other hand, no ettringite was found. The C-S-H phase provides the highest strength of mortars made from cement with this clinker after 48 h of hydration. In the cement paste containing C1 clinker, ettringite is found in the form of thin, long, and interconnecting needles at the observed points. Ettringite creates the crystalline skeleton responsible for the early strength of mortars made with this cement [8,10,16], but a weaker microstructure than that derived from C-S-H [16,44]. It results in a lower compressive strength of mortar made from cement containing this clinker than mortar containing cement from C2 clinker. In all samples there are non-hydrated cement particles covered with hydration products. At the observed points, the sample made of cement containing C1 clinker has single crystals of portlandite (Ca(OH)_2_) and cement grains covered with C-S-H phase.

#### 3.2.2. Cement Pastes Modified with Crystallization Seeds (CS)

**Figure 7:**
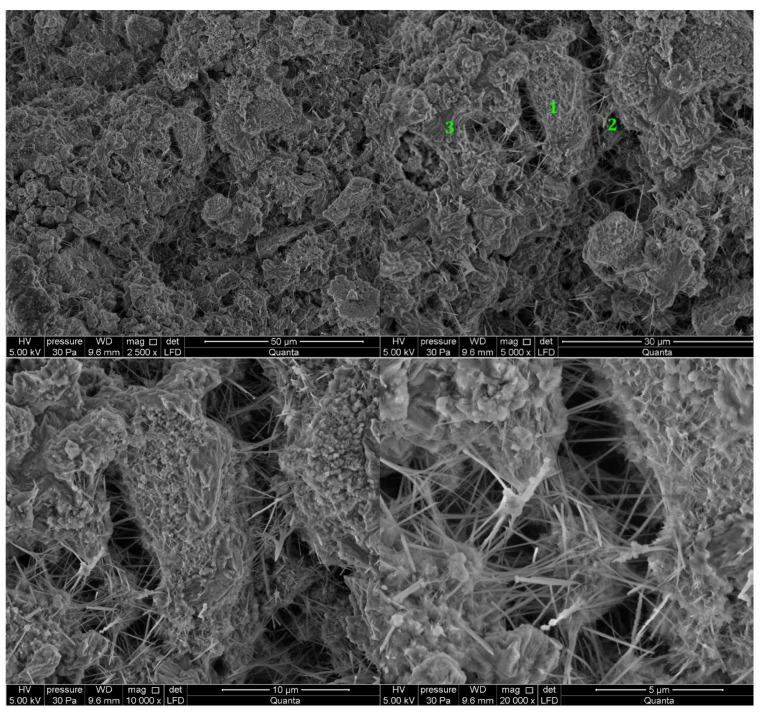
SEM images for cement paste made with CEM II/B-S manufactured with clinker C1, modified with crystallization seeds (CS). 1—C-S-H phase, 2—ettringite, 3—calcite.

**Figure 8:**
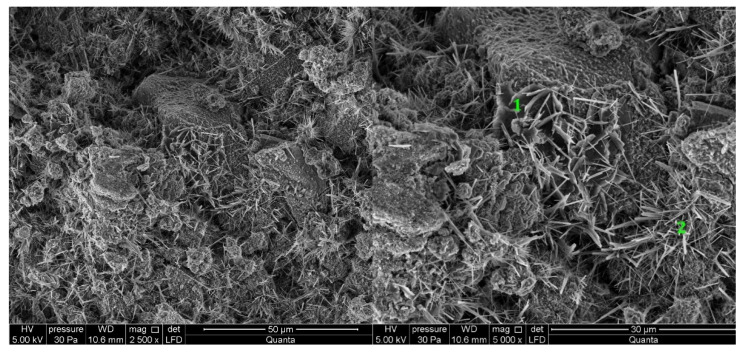

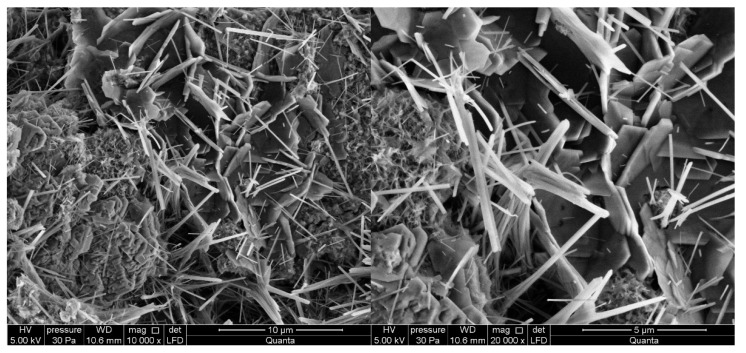
SEM images for cement paste made with CEM II/B-S manufactured with clinker C2, modified with crystallization seeds (CS). 1—AFm phase, 2—ettringite.

The cement paste samples modified with an activator containing crystallization seeds (CS) exhibits the presence of C-S-H type (II) phase in the form of honeycomb, ettringite, which is present in the form of long, thin, and interconnecting rods and single crystals of portlandite in the examined observation points in the case of cement containing C1 clinker (Figure 7). Higher content of C-S-H was found compared to the observed points of the reference sample, which corresponds to the results presented in the previous research [56]. In the case of a paste containing C2 clinker cement modified with CS, the observed points reveal a C-S-H phase in the form of a honeycomb. In contrast to the reference sample, ettringite is present in the sample in the form of thicker bars, in contrast to the reference sample. Single crystals of portlandite and AFm phase are present (Figure 8). This clinker is characterized by a higher amount of C_3_S phase (69%) and C_2_S phase (12%). The total content of silicate phases is higher (81%) than that of C1 clinker (75%), and the content of aluminate phases is lower (C2—16%, C1—21%). Thus, it can be concluded that the efficiency of crystallization seeds (CS) increases with increasing content of C_3_S and C_2_S phases and decreasing content of C_3_A and C_4_AF phases. The results are consistent with other research provided for OPC without mineral additives [57] and C_3_S paste [58]. Present research gives the background for further investigation of nucleation seeds action in cement containing supplementary cementitious materials (SCMs) like GGBFS or fly ash.

#### 3.2.3. Cement Pastes Modified with Calcium Nitrate (CN)

**Figure 9:**
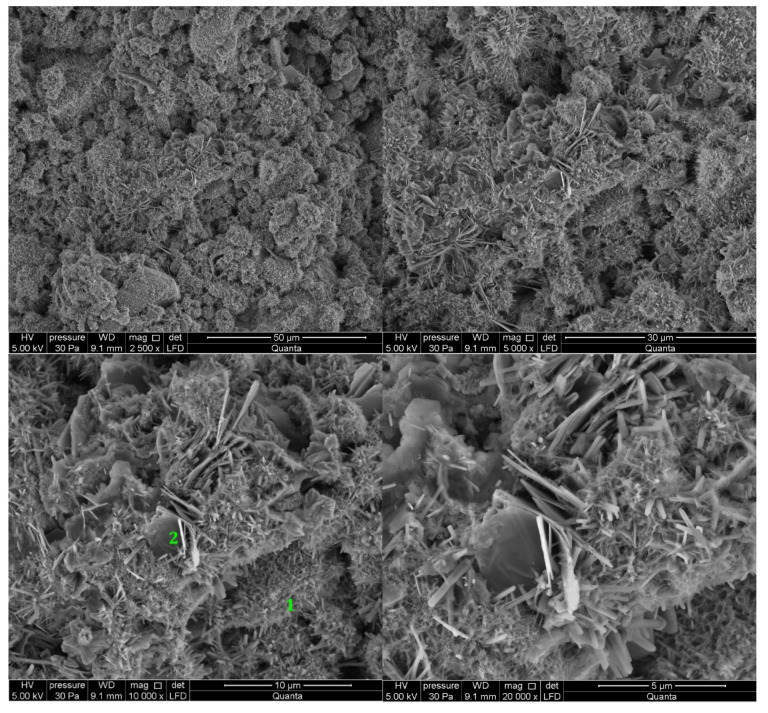
SEM images for cement paste made with CEM II/B-S manufactured with clinker C1, modified with calcium nitrate (CN). 1—C-S-H phase, 2—portlandite.

**Figure 10:**
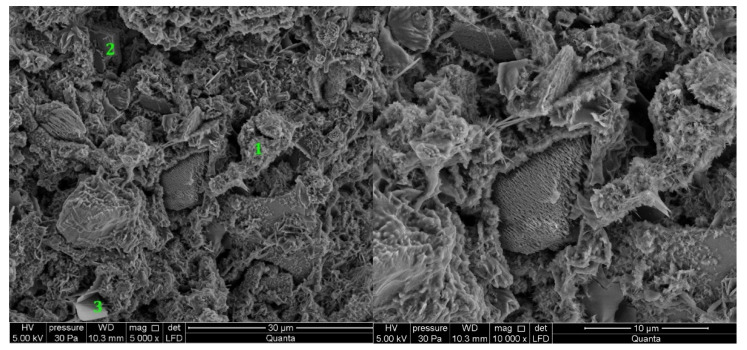

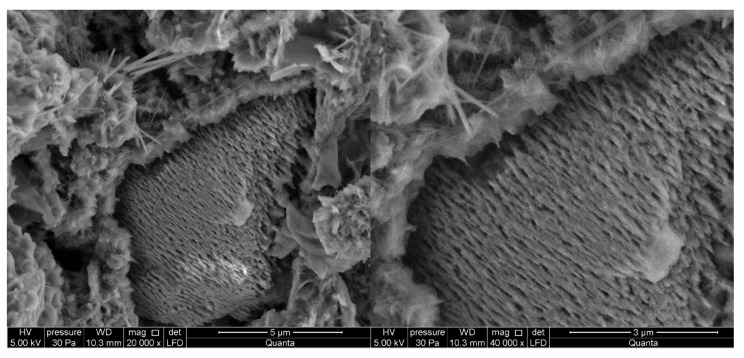
SEM images for cement paste made with CEM II/B-S manufactured with clinker C2, modified with calcium nitrate (CN). 1—C-S-H, 2—CaO, 3—portlandite.

In the case of the modification of slurries made with calcium nitrate (CN), it can be observed that the morphology of the C-S-H phase does not differ in the tested sample areas. The C-S-H phase is still in the form of a honeycomb. In the observed spots, the cement paste sample containing C1 clinker shows fewer ettringite crystals, which are smaller in length than to the sample that was not modified with calcium nitrate (CN) (Figure 9). In the case of cement containing C2 clinker, ettringite is not present in the observed points after calcium nitrate (CN) modification as much as in the case of unmodified cement (Figure 10). The presence of CaO crystal in the newly hydrated paste is a manifestation of the presence of free CaO in Portland clinker (Table 1). It is called dead-burn lime or sintered lime. The existence of free CaO in cement is the result of clinker production at 1450 °C. At this temperature, a thin, insoluble layer is formed on its surface, slowing its reaction with water and forming Ca(OH)_2_ [59,60,61]. It is a significant difference compared to quicklime, produced at temperatures up to 1050 °C and reacts with water without delay. Despite slight differences in the microstructure of unmodified and calcium nitrate (CN)-modified hardened cement pastes made from different clinkers, the compressive strength of mortars modified with calcium nitrate (CN) is higher than those not modified. It is stated that CN influences both C_3_S and C_3_A phase reactions. The AFm containing nitrates is formed from the latter. Accelerated C_3_S hydration results in tighter microstructure and better mechanical performance [62]. The C_3_S hydration products (especially C-S-H phase) have the same atomic structure with and without CN [26]. The present research results show that CN is effective regardless of the clinker composition—reacting both with silicate and aluminate phases—and also with GGBFS cements.

#### 3.2.4. Cement Pastes Modified with Sodium Hydroxide (NaOH)

**Figure 11:**
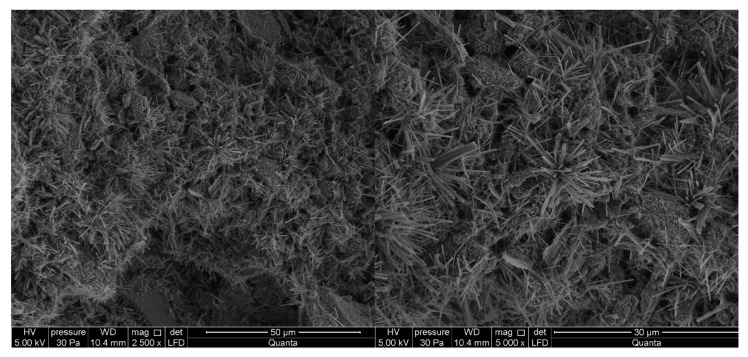

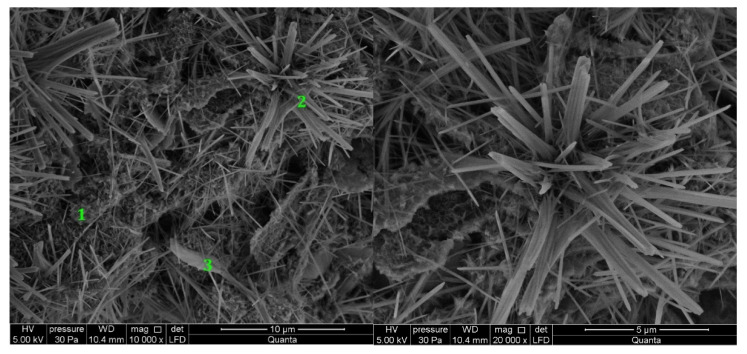
SEM images for cement paste made with CEM II/B-S manufactured with clinker C1, modified with sodium hydroxide (NaOH). 1—C-S-H phase, 2—ettringite, 3—portlandite.

**Figure 12:**
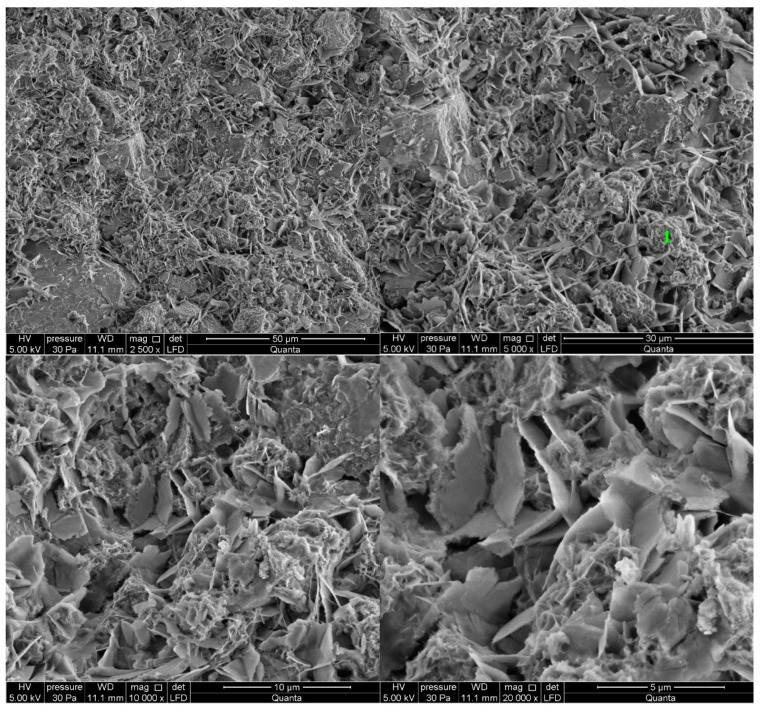
SEM images for cement paste made with CEM II/B-S manufactured with clinker C2, modified with sodium hydroxide (NaOH). 1—C-S-H phase.

After two days of curing, sodium hydroxide (NaOH) significantly reduces the compressive strength of mortars. This is applicable to cement containing both clinkers. In the case of cement made from clinker C1, the observed ettringite crystals are thicker and shorter (Figure 11), making the spatial structure less developed [8,10,16]. The morphology of the C-S-H phase and the portlandite (Ca(OH)_2_) content of the observed points are similar to those of the reference sample. In cement paste sample made from C2 clinker, the C-S-H phase is present in the form of a honeycomb and single crystals of portlandite are present. Ettringite is not present in the analyzed areas of these samples (Figure 12). Sodium hydroxide compromises the compressive strength also in early terms. The results are similar to those obtained for white Portland cement also in terms of microstructure. NaOH lowers the total amount of hydration products, especially the ettringite [63].

#### 3.2.5. Cement Pastes Modified with Cement Kiln Dust (CKD)

**Figure 13:**
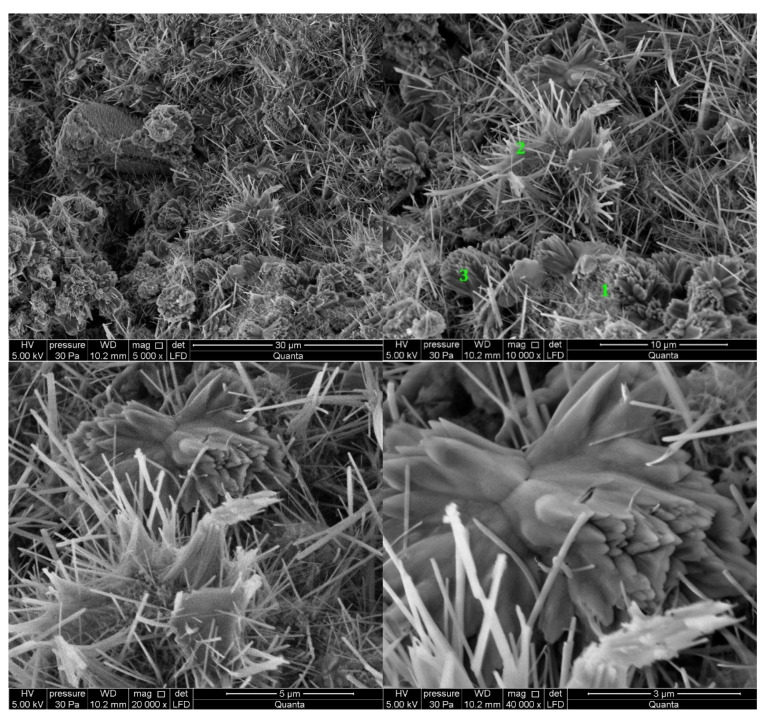
SEM images for cement paste made with CEM II/B-S manufactured with clinker C1, modified with cement kiln dust (CKD). 1—C-S-H phase, 2—ettringite, 3—calcite.

**Figure 14:**
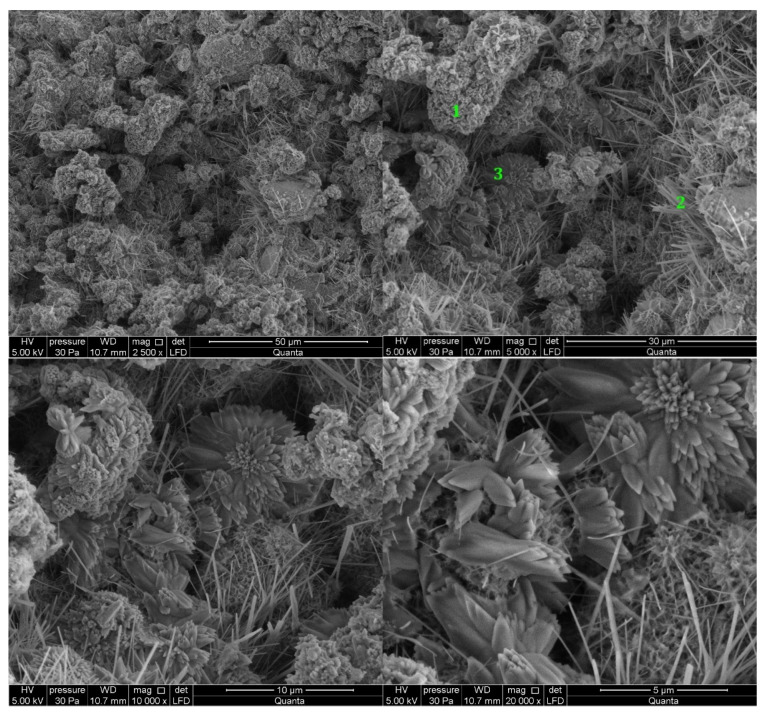
SEM images for cement paste made with CEM II/B-S manufactured with clinker C2, modified with cement kiln dust (CKD). 1—C-S-H phase, 2—ettringite, 3—calcite.

The microstructure of cement kiln dust (CKD)-modified pastes is similar to pastes without CKD, regardless of the type of clinker used to make the cement (Figure 13 and Figure 14). The difference for the analyzed areas of all samples is the presence of calcite in the form of rosettes. Its occurrence is related to the large amount of carbonates contained in CKD in the form of ignition losses. Ettringite, which did not appear in the reference sample, can be observed in the observed points of the sample of paste made from C2 clinker cement with the addition of CKD (Figure 14). The similarity to the microstructure of unmodified pastes is due to the similar dust composition of CKD and cement [64,65]. CKD usefulness is backed up also by its cooperation with GGBFS as an alkali activator for materials with latent hydraulic properties [66]. Some research reports that the density of the cement matrix is also enhanced [34].

### 3.3. X-ray Diffraction (XRD) Analysis

C-S-H phase is the main component of the hardened Portland cement paste in terms of both amount and influence on the strength evolution. Researchers have different opinions on whether its structure is amorphous or semi-crystalline (also poorly-crystalline or nanocrystalline) [67,68,69]. This crystallinity of the product depends on the conditions during its formation and Ca/Si ratio [70,71,72,73]. In the early hydration stage, the second amorphous component of Portland cement–GGBFS blends is GGBFS itself [8,74,75,76]. However, those two components are very hard to be distinguished in XRD analysis because the nature of both is non-crystalline.

**Figure 15:**
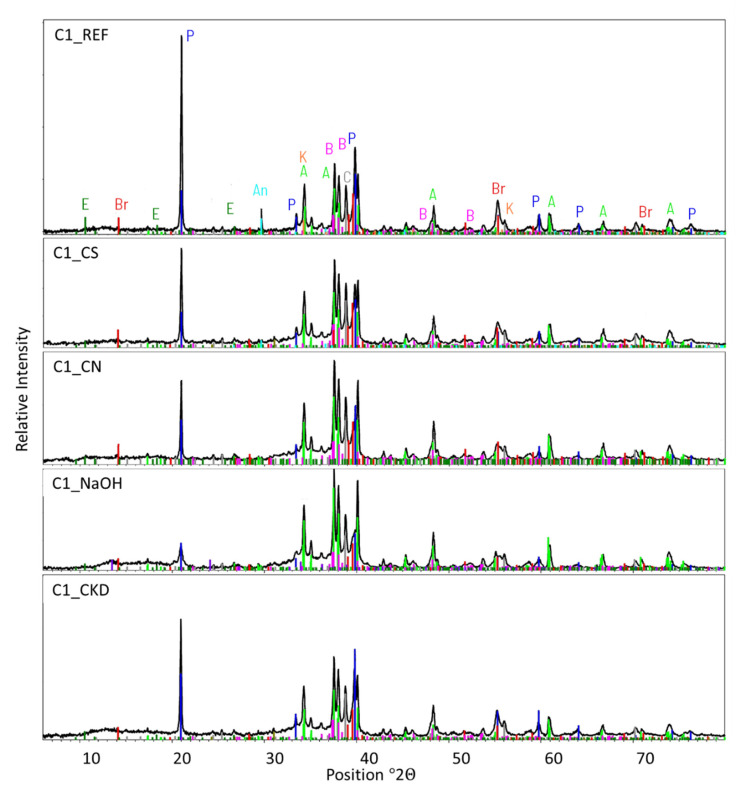
XRD patterns for cement pastes made with CEM II/B-S manufactured with clinker C1, non-modified and modified with accelerating admixtures.

The XRD analysis results are presented in Table 5 and Figure 15. These indicate that the smallest amount of the amorphous phase (55.1%) is present in the cement paste sample without modifications. Pastes containing crystallization seeds, calcium nitrate, and CKD show a similar quantity (61.1–62.3%). The largest amount of amorphous phase is present in NaOH-modified paste (68.1%). More amorphous phase means that more C-S-H phase is formed from Portland cement hydration. In addition, the amorphous non-hydrated GGBFS forms the amorphous C-S-H phase as well. Thus, the increase of total amorphous phase content indicates a faster hydration reaction rate for all modified pastes, at the greatest extent for NaOH-modified paste. 

The second hydration reaction product is calcium hydroxide (Ca(OH)_2_, portlandite). Its amount is the highest in non-modified paste (9.2%) and lowest in pastes modified with CN and NaOH (4.4%) (Table 5, Figure 15). The lower amount of portlandite in CN-modified paste was also reported by other researchers [22,62]. Medium values (6.5% and 8.5%, respectively) is observed for CS- and CKD-modified pastes. GGBFS shows both latent hydraulic and pozzolanic properties [77,78]. The portlandite is consumed during the pozzolanic reaction between it and GGBFS resulting in C-S-H phase formation. Thus, the lower amount of portlandite is evidence of a faster reaction of GGBFS.

The lower amount of non-hydrated calcium silicate components (C_2_S, C_3_S) of cement are present in all modified pastes than in the reference one. Amount of aluminate (C_3_A) phase is similar in non-modified pastes and ones modified with CS and CN (5.4–5.9%). However it is significantly lower for pastes modified with CKD and NaOH admixture (3.6% and 3.2%, respectively). Ferrite (C_4_AF) phase content is similar in all pastes (2.0–2.3%). Taking above into account together with starting amount of those components in cement paste (considering clinker/GGBFS ratio)—C_2_S = 6.6%, C_3_S = 38.4%, C_3_A = 8.4%, C_4_AF = 4.2%—generally lower content of non-hydrated compounds is the evidence of faster hydration reaction for pastes modified with all admixtures. The results also imply that while all admixtures accelerate the hydration of calcium silicates, only NaOH and CKD influences the rate of aluminate phase reaction.

Because of a limited number of XRD results for cement pastes modified with accelerating admixtures, especially for modern accelerators, it is a promising path for researchers to follow.

### 3.4. Mercury Intrusion Porosimetry (MIP) Analysis

**Figure 16:**
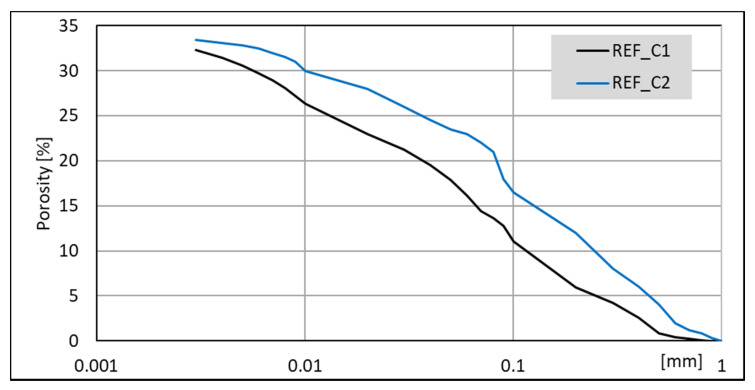
MIP cumulative results for non-modified cement pastes made with CEM II/B-S manufactured with clinkers C1 and C2.

The porosimetry tests were performed for all pastes. Results for non-modified pastes (Figure 16) indicates that lower porosity is generated in C1 clinker. Moreover, it contains more C_3_A and less C_2_S and C_3_S together than C2 clinker. Summary pore volume is similar (33 –34%), but C1-based paste exhibits greater amount of larger pores.

**Figure 17:**
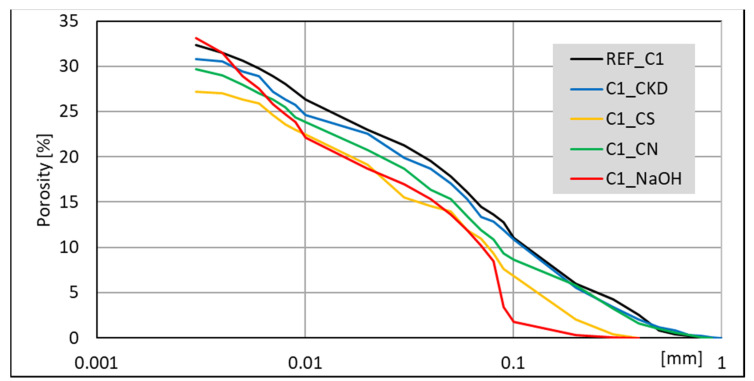
MIP cumulative results for cement pastes made with CEM II/B-S manufactured with clinkers C1, modified with accelerating admixtures.

**Figure 18:**
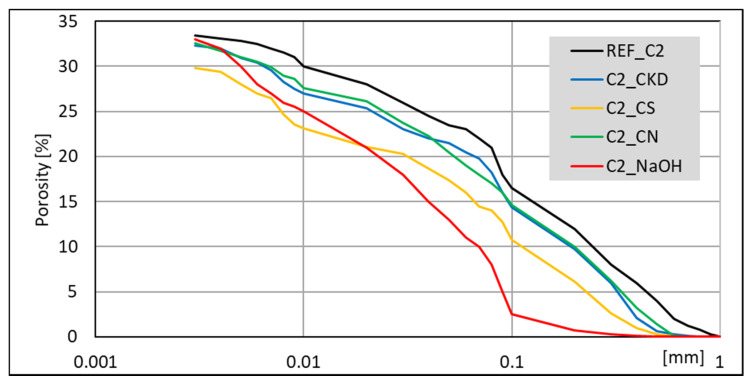
MIP cumulative results for cement pastes made with CEM II/B-S manufactured with clinkers C2, modified with accelerating admixtures.

Pastes modified with accelerating admixtures, generally show lower or similar total pore volume, independent of the clinker used to manufacture the cement (Figure 17 and Figure 18). The lowest total pore volume show pastes modified with CS admixture (27% for clinker C1 and 30% for clinker C2). The porosimetry results are consistent with compressive strength (Figure 3 and Figure 4). However, it is necessary to mention that CN-modified paste exhibits subtly greater porosity, but very similar strength. Results of SEM visual analysis also shows more tight structure (Figure 9 and Figure 10). Modification of pastes with NaOH for both cements led to the greatest porosity from the modified pastes. However, it resulted in similar total porosity to the non-modified one. It is also consistent with the compressive strength of respective mortars—the strength of NaOH-modified ones was the lowest from all tested samples. Although the compressive strength of reference mortar is significantly higher, the porosity cannot explain their differences. This issue needs to be subjected to further research. Visual observations also show greater porosity of reference C2 paste (Figure 6) and NaOH-modified one with C1 clinker (Figure 11) smaller pores. It is the result of a high amount of ettringite crystals, but with a greater volume.

Results obtained for CN and CKD modifications of C1 clinker based cement are in the middle (30% and 31%, respectively). Those for C2 clinker-based cement are similar to total pore volume for the non-modified one and that modified with NaOH. The lower porosity of CKD-modified pastes than reference paste is connected to its fineness and very high specific surface area. The compressive strength of CKD containing mortar is also higher than for the reference one. The microstructure of C-S-H phase and ettringite is also finer for pastes with this modification (Figure 13 and Figure 14). The CKD influence on the porosity of cement composites is reported to be different—depending on the source it increases [79] or decreases [33,34]. The effect of CKD on microstructure and factors affecting its various behavior requires further research. Calcium nitrates influence on the pore structure of cement pastes is reported to be similar to this observed in current research–both pore diameter and total volume tend to decrease [80,81]. Article [81] also reports that NaOH-modified mortars’ porosity exhibited similar porosity to non-modified ones.

It is worth noting that while the volume of pores with different dimensions is proportional between non-modified and modified with CS, CN, and CKD pastes, the porosity of NaOH-modified pastes is different. Therefore, those pastes, made from both clinkers, exhibit a smaller amount of large pores (<0.1 mm).

## 4. Conclusions

Mortars made from cement containing clinker with a higher tricalcium silicate content (C2 clinker) have the highest compressive strength after 48 h of curing. This was caused by the faster development of the C-S-H phase structure in cement with high C_3_S content.

Mortars made from clinker C1, with a higher tricalcium aluminate content, achieved slightly lower compressive strengths after this time. In this case, the developed C-S-H phase structure, but mainly the ettringite, which forms a crystalline skeleton composed of long and thin interconnecting crystals, are responsible for the compressive strength. The ettringite formation is promoted by high C_3_A content in the cement. All accelerators, except NaOH, enhanced the early compressive strength.

SEM and EDS examination of the microstructure of cement pastes modified with accelerating admixtures at the observed points did not reveal differences that would be sufficient to explain the changes in compressive strength caused by the application of setting and hardening accelerators after two days of curing. However, it should be noted that the observed differences in intermeshed ettringite needles and C-S-H phase patches are qualitative and not quantitative. Still, those are not sufficient to explain differences in strength properties. The microstructure of CKD-modified pastes is similar to pastes without CKD (because of the similar CKD and OPC composition), but contains calcite in the form of rosettes (caused by the large amount of carbonates in CKD). Accelerator containing crystallization seeds (CS) caused better development of C-S-H phase and change in the ettringite to form thicker bars. The efficiency increases with content of calcium silicate phases. After modification with calcium nitrate, the morphology of the C-S-H phase is similar, but fewer and shorter ettringite crystals occurs. With NaOH presence in the cement paste, the ettringite crystals are thicker and shorter, making the spatial structure less developed (in C1 paste) and it is not present at all in the C2 paste.

The lower amount of portlandite shown in XRD analysis is the proof of faster reaction of GGBFS in the presence of accelerators.

The increase of total amorphous phase content and lower non-hydrated compounds content indicate the faster hydration reaction rate for all accelerating admixture-modified pastes. The latter indicates also that all admixtures accelerate the hydration reaction of calcium silicate phases of cement, but only NaOH and CKD influence the rate of aluminate phase reaction.

The total pores volume is independent of the cement phase composition, while the pore size distribution is not. The lowest total pore volume was observed for pastes modified with CS admixture. The porosity results support differences in compressive strength. NaOH modification of cement pastes results in the smaller dimension of pores, while the total volume remains similar. The lower porosity of pastes with CKD is connected to its fineness and very high specific surface area.

## Figures and Tables

**Table 1:** Chemical and phase composition of Portland clinkers, GGBFS, and anhydrite, mass %.

Sym.	SiO_2_	Al_2_O_3_	Fe_2_O_3_	CaO	MgO	SO_3_	Cl^–^	Na_2_O	K_2_O	CaO_free_	Ign. Loss.
C1	20.25	6.83	3.23	65.66	1.39	0.69	0.01	0.15	1.02	2.80	0.15
C2	21.25	5.00	3.40	64.81	2.09	0.55	0.03	0.11	1.03	1.48	0.35
GGBFS	37.35	7.30	1.22	43.90	5.73	0.62	0.03	0.55	0.56	-	0.17
Anhydrite	0.61	-	-	40.16	0.40	54.83	-	0.02	-	-	2.71
	**Phase Composition**							**Blaine’s Specific Surface Area**			
	**C_2_S**		**C_3_S**		**C_3_A**		**C_4_AF**				
C1	11		64		14		7	3000 cm^2^/g			
C2	12		69		4		12	3000 cm^2^/g			
GGBFS	-		-		-		-	3900 cm^2^/g			

**Table 2:** Chemical composition of CKD, mass %.

SiO_2_	Al_2_O_3_	Fe_2_O_3_	CaO	MgO	SO_3_	Cl^–^	Na_2_O	K_2_O	P_2_O_5_	TiO_2_	Mn_2_O_3_	SrO	ZnO	Ign. Loss.
18.16	4.55	2.03	57.13	1.26	1.95	1.44	0.12	2.38	0.12	0.22	0.06	0.10	0.24	11.70

**Table 3:** Composition of mortars for compressive strength tests.

No.	Portland Clinker		GGBFS	Anhydrite	Sand	Water	CKD	Admixture		
	type	[g]						type	[% m.c.]	[g]
1	C1	285.3	153.6	11.07	1350	225	-	-	-	-
2		285.3	153.6	11.07	1350	225	-	CS	4.0	18.0
3		285.3	153.6	11.07	1350	225	-	CN	2.0	9.0
4		285.3	153.6	11.07	1350	225	-	NaOH	2.0	9.0
5		270.9	145.8	10.8	1350	225	22.5	-	-	-
6	C2	284.8	153.4	11.8	1350	225	-	-	-	-
7		284.8	153.4	11.8	1350	225	-	CS	4.0	18.0
8		284.8	153.4	11.8	1350	225	-	CN	2.0	9.0
9		284.8	153.4	11.8	1350	225	-	NaOH	2.0	9.0
10		270.4	145.6	11.5	1350	225	22.5	-	-	-

**Table 4:** Composition of cement pastes for SEM, EDS, XRD (for C1), and porosimetry and initial setting time (for C1 and C2) tests.

No.	Portland Clinker		GGBFS	Anhydrite	Water	Water Demand	CKD	Admixture		
	type	[g]			[g]	%	[g]	type	[% m.c.]	[g]
1	C1	285.3	153.6	11.07	217	43	-	-	-	-
2		285.3	153.6	11.07	218	44	-	CS	4.0	18.0
3		285.3	153.6	11.07	169	34	-	CN	2.0	9.0
4		285.3	153.6	11.07	183	37	-	NaOH	2.0	9.0
5		270.9	145.8	10.8	167	33	22.5	-	-	-
6	C2	284.8	153.4	11.8	149	30	-	-	-	-
7		284.8	153.4	11.8	150	30	-	CS	4.0	18.0
8		284.8	153.4	11.8	150	30	-	CN	2.0	9.0
9		284.8	153.4	11.8	178	36	-	NaOH	2.0	9.0
10		270.4	145.6	11.5	152	30	22.5	-	-	-

**Table 5:** Quantitative XRD analysis results.

Paste	C1_REF	C1_CS	C1_CN	C1_NaOH	C1_CKD
	Content [%]				
C3S	16.1	13.3	14.6	14.1	14.9
C2S	9.6	8	8.8	6.8	7.8
C3A	5.4	5.9	5.6	3.2	3.6
C4AF	2.6	2.3	2.1	2.0	2.2
Portlandite	9.2	6.5	4.4	4.4	8.5
Anhydrite, calcite, quartz, ettringite, other	Each < 1.0				
Sum of crystalline components	42.9	36.0	35.5	30.5	37.0
Amorphous phase	55.1	62.2	62.3	68.1	61.1

## Data Availability

Not applicable.
